# Keratinocytes as Depository of Ammonium-Inducible Glutamine Synthetase: Age- and Anatomy-Dependent Distribution in Human and Rat Skin

**DOI:** 10.1371/journal.pone.0004416

**Published:** 2009-02-10

**Authors:** Lusine Danielyan, Sebastian Zellmer, Stefan Sickinger, Genrich V. Tolstonog, Jürgen Salvetter, Ali Lourhmati, Dieter D. Reissig, Cristoph H. Gleiter, Rolf Gebhardt, Gayane Hrachia Buniatian

**Affiliations:** 1 Department of Clinical Pharmacology, University Hospital of Tübingen, Tübingen, Germany; 2 Institute of Biochemistry, Medical Faculty, University of Leipzig, Leipzig, Germany; 3 Heinrich-Pette-Institute for Experimental Virology and Immunology, Hamburg, Germany; 4 Institute of Anatomy, University of Leipzig, Leipzig, Germany; Tufts University, United States of America

## Abstract

In inner organs, glutamine contributes to proliferation, detoxification and establishment of a mechanical barrier, i.e., functions essential for skin, as well. However, the age-dependent and regional peculiarities of distribution of glutamine synthetase (GS), an enzyme responsible for generation of glutamine, and factors regulating its enzymatic activity in mammalian skin remain undisclosed. To explore this, GS localization was investigated using immunohistochemistry and double-labeling of young and adult human and rat skin sections as well as skin cells in culture. In human and rat skin GS was almost completely co-localized with astrocyte-specific proteins (e.g. GFAP). While GS staining was pronounced in all layers of the epidermis of young human skin, staining was reduced and more differentiated among different layers with age. In stratum basale and in stratum spinosum GS was co-localized with the adherens junction component ß-catenin. Inhibition of, glycogen synthase kinase 3β in cultured keratinocytes and HaCaT cells, however, did not support a direct role of ß-catenin in regulation of GS. Enzymatic and reverse transcriptase polymerase chain reaction studies revealed an unusual mode of regulation of this enzyme in keratinocytes, i.e., GS activity, but not expression, was enhanced about 8–10 fold when the cells were exposed to ammonium ions. Prominent posttranscriptional up-regulation of GS activity in keratinocytes by ammonium ions in conjunction with widespread distribution of GS immunoreactivity throughout the epidermis allows considering the skin as a large reservoir of latent GS. Such a depository of glutamine-generating enzyme seems essential for continuous renewal of epidermal permeability barrier and during pathological processes accompanied by hyperammonemia.

## Introduction

Glutamine synthetase (GS; EC 6.3.1.2) is the only known human enzyme capable of catalyzing glutamine (Gln) synthesis from ammonia and glutamate and, thereby, contributes to multiple tissue events regulated by this amino acid. Gln represents the most abundant amino acid in mammalian blood reflecting its function as the major shuttle of amino-nitrogen between cells. It plays an important role in acid-base homeostasis and serves as a fuel source for many types of cells. In rapidly dividing cells GS is required as a precursor for the synthesis of multiple biologically active compounds including purines, pyrimidines, and aminosugars [1]. Last but not least, Gln is a major constituent of proteins particularly in skin.

GS is found in many, if not all organs [2]. However, in most of them, expression of GS is confined to specific cell populations only. Prominent examples for this are the pericentral hepatocytes in liver [3], [4] and certain cells lining the proximal convoluted tubules in kidney [2], [5], [6]. In addition, GS at a lower but highly adaptive level is expressed in almost every organ in glial fibrillary acidic protein (GFAP)-producing perivascular cells that play an important role in tissue homeostasis at the blood-tissue interface [7]. These cells comprise astrocytes located at the blood brain barrier [8], activated hepatic stellate cells (HSC) at the blood-tissue interface in liver [9], and Leydig cells of the testis [10]. In pathological conditions, on the other hand, GS may be expressed in cells that normally do not produce the enzyme, such as neurons in Alzheimers disease. [11], [12], [13]. Thus, the cellular expression of GS in each organ is highly specific and has to be characterized by immunocytochemistry or similar techniques, in order to draw conclusions on its precise role and function.

Compared to many other organs, GS activity in rat skin has been found to be rather moderate [14], [15]. Consequently, interest in GS in skin remained low for a long time and this organ was not even mentioned in a comprehensive survey on GS in murine organs [16]. Even less is known so far about the cellular distribution as well as the specific function of GS in this organ. However, recent observations on humans with inherited GS deficiency convincingly showed the importance of GS for skin-specific functions. The consequences of this metabolic disease were illustrated by severely disturbed epidermal development, rapid appearance of focal erythema and blistering of the integumentum resulting in early postnatal death [17], [18]. These findings concerning integrity, regeneration and molecular characteristics of human and rodent skin propose a previously unrecognized considerable demand for Gln in the developing skin and an important local function of GS in skin integrity. An increased need for Gln during skin regeneration was also suggested by the finding that major burn injury of skin leads to the induction of GS expression in specific tissues such as lung, muscle, kidney and liver [19]. Likewise, thermal injury of 33–35% of body surface area is accompanied by increased levels of Gln in muscle, skin and adipose tissue preparations [20]. These studies call for detailed investigation of distribution of GS in different cell types and structures of the skin as well as mechanisms involved in the regulation of GS levels in keratinocytes.

Multiple structural and functional peculiarities and specific anatomy of the skin, offered an interesting object for further examination of our hypothesis concerning commonness of mechanisms operating at the border of different environments and tissues, i.e., blood-tissue, blood-urine and air-tissue interfaces. The co-localization in skin cells of several astrocyte-specific antigens [21] considerably strengthen our viewpoint concerning the universality of mechanisms regulating the homeostasis in different organs. This raised the question of whether the peculiar ability of smooth muscle alpha-actin (SMAA) and GFAP-containing astrocytes and HSC to express GS in a dynamic fashion contributing to tissue homeostasis and protection [7], [9], [22]–[24] may be found as well in epidermal keratinocytes forming part of the tissue-air interface.

To learn more about the skin-specific functions of GS we have investigated in detail the distribution of GS in different cell types and structures in human and rat skin samples of different age. In accordance with studies demonstrating a dependency of the antigenic composition of skin cells on their anatomy and age [25], [26], the specific cellular expression and localization of GS in different skin samples of humans and rats showed some variation depending on the age of the donor and on the location from which the skin sample was taken. These variations, for which we provide some prominent examples for different parts of skin from children and adult humans as well as from newborn and adult rats, indicate a close association of the specific cellular localization of GS with different functional and structural features of skin. In order to emphasize this association and, at the same time, to illustrate possible exceptions, we performed co-localizations of GS with each of the marker proteins previously shown to characterize these functional and structural differences [21]. Furthermore, we aimed at elucidating basic mechanisms involved in the regulation of GS levels in skin keratinocytes. In the light of recent in vitro studies demonstrating the influence of Gln on establishment of adherens and tight junctions complexes [27]–[30] and of the novel role of wingless-type mouse mammary tumour virus integration site family member (Wnt)/β-catenin signaling in regulating GS expression in liver [31], a series of investigations has been performed to examine the relation between GS and β-catenin. Our results suggest multiple roles of GS in maintaining barrier structure and function of skin supported by novel specific regulatory features.

## Results

### Cellular localization of GS in skin

#### Distribution of GS and its co-localization with GFAP and SMAA in human skin slices

**Figure 1:**
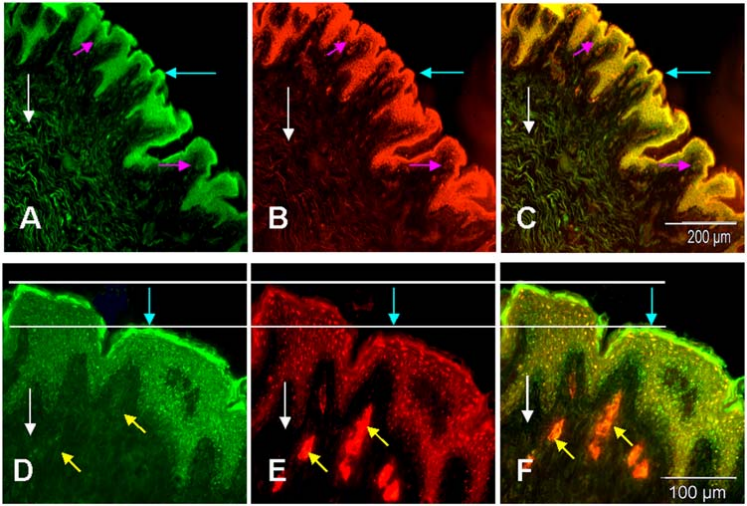
Presence of GS in human foreskin of children and regional variations in its distribution revealed in co-localization studies with GFAP, or with SMAA. (A–D) Expression of GS (A) and GFAP (B) and merged micrograph of GS and GFAP (C) immune reaction in human foreskin sections. (D–F) Expression of GS (D) and SMAA (E) and merged micrograph of GS and SMAA immune reaction (F) in a human skin section. The line connecting the D–F clearly shows strong expression of SMAA (E) in stratum granulosum and its absence of stratum corneum. The latter is strongly stained for GS (D). GS and SMAA merge at the interface of stratum corneum boarder and stratum granulosum (F). Binding of specific antibodies was visualized with FITC-conjugated goat anti-rabbit IgG and Cy3-conjugated goat anti-mouse IgG. Blue arrows point to stratum corneum, magenta arrows to stratum basale white arrows to fibroblasts and yellow arrows to small and middle-sized vessels. Scale bar: A–D, H 200 μm; E–G 100 μm.

Double labeling of sections of human foreskin with polyclonal antibodies (pAb) directed against GS (green, Figure 1A) and monoclonal antibodies (mAb) against bovine GFAP (red, Figure 1B) revealed almost complete co-localization of both proteins in all layers of the epidermis: stratum basale (magenta arrows throughout Figure 1), stratum spinosum, stratum granulosum and stratum corneum (blue arrows throughout Figure 1). Similar spatial distribution of GS and GFAP was illustrated by intense yellow staining of epidermis in merged micrographs (Figure 1C). In young foreskin, the stratum corneum is very thin. The reaction of GS was particularly strong in stratum granulosum and stratum corneum and slightly weaker in stratum basale and stratum spinosum. In the dermis of these skin samples (white arrows), GS was expressed weaker than in epidermis (cf. structures indicated by blue and white arrows in Figures 1A, C). Similar findings for GS were obtained also in older foreskin (not shown).

In skin sections double-labeled with antibodies against GS and anti-SMAA, occasionally enhancement of GS staining in the nuclear area of the keratinocytes located in inner layers of the epidermis could be seen (Figure 1D). In accordance with our recent study [21], SMAA (Figure 1E, red) was preferentially localized in the perinuclear and nuclear area of human foreskin keratinocytes resulting in yellow or orange staining of this area in merged micrographs (Figure 1F). The line drawn through Figures 1D, E, F, clearly demarcates the highest accumulation of SMAA expressing cells in the stratum granulosum and its absence in stratum corneum of young human foreskin (Figure 1E) which strongly stained for GS (Figure 1D). GS and SMAA were both present in stratum granulosum and inner parts of epidermis (Figure 1F). In foreskin dermis, on the other hand, SMAA (red, Figure 1E) could barely be detected in fibroblasts moderately and evenly expressing GS (green, Figures 1A, C, D, F). Instead, it was strongly expressed in myofibroblasts surrounding the middle-sized vessels (yellow arrows in Figure 1E, F) and in the papillary zone of the dermis in which GS was poorly presented (orange staining in merged Figure 1F). GS and SMAA were also co-localized in association to large vessels situated in the reticular part of the dermis (not shown).

#### Co-localization of GS and β-catenin in aged human temple skin

**Figure 2:**
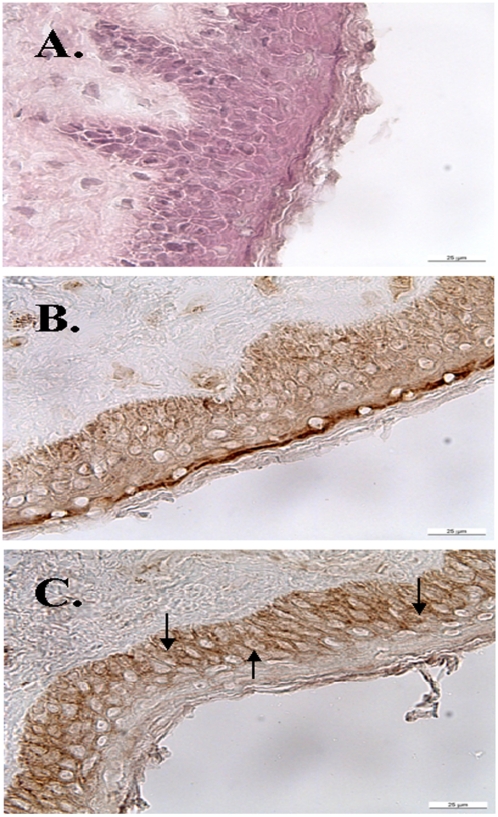
Distribution of GS and β-catenin in normal senile human temple skin. (A) hematoxiline staining of a skin sample. Stratum basale can be identified by cells with prominent nuclei. (B) GS is localized throughout the viable part of the epidermis. The highest staining intensity can be detected at the interface of stratum granulosum and stratum corneum. (C) Localization of β-catenin in lower parts of the epidermis. β-catenin shows a strong membraneous and cytoplasmic staining in stratum basale and in stratum spinosum. Arrows in (C) point to the presence of catenin in the nucleus of keratinocytes. Scale bar 25 μm.

Since the Wnt/ß-catenin signalling pathway was recently found to play an important role in the regulation and zonal expression of GS in liver [31], [32] we wondered whether ß-catenin might be co-localized with GS in skin as well. Figure 2A shows conventional eosin staining of temple skin of aged (70-year-old) male patient. Immunohistological staining of this region of aged skin with a monoclonal anti-GS-antibody and peroxidase (POD)-coupled secondary antibodies revealed the presence of GS in epidermis (Figure 2B). In aged human temple skin, the staining for GS was especially strong in stratum granulosum, while it was almost absent from the utmost layer of stratum corneum. β-catenin, was relatively strongly expressed in cells of stratum basale and stratum spinosum (Figure 2C). In stratum granulosum the intensity of β-catenin staining abruptly decreased. Some keratinocytes expressed β-catenin preferentially in the membranes and slightly in the cytoplasm but not in the nucleus, whereas in other keratinocytes a faint staining of the nuclei could be observed. The heterogeneity in staining for β-catenin and its intercellular localization may be explained by differences in the individual rates of differentiation of keratinocytes, as well as by region-specific requirements. Remarkably, GS (at a moderate level) and β-catenin were both present in stratum basale, the actual proliferative compartment, and in stratum spinosum.

#### Age-specific and regional distribution of GS and GFAP in rat skin

**Figure 3:**
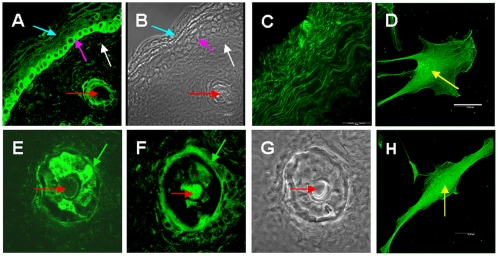
Expression of GS in newborn rat scalp sections CLS microscopy of skin preparations stained for GS. (A) Representative of serial optical sections obtained by CLS microscopy of newborn rat skin stained for GS (green). (B) Phase contrast micrograph of the field shown in (A). (C) Wide-field fluorescence microscopy of fibroblasts in human skin. (D, H) Representative of serial optical sections obtained by CLS microscopy of cultured human fibroblasts stained for GS. GS was detected using rabbit antiserum and FITC-conjugated goat anti-rabbit IgG. (E, F) Representatives of serial optical sections obtained by CLS microscopy of hair follicle in newborn rat skin section stained for GS. (G) Phase contrast micrograph of the field shown in E and F. Blue arrows point to epidermis, magenta arrows to the keratinocytes forming the stratum basale of the epidermis, white arrows to fibroblasts, green arrows to the outer sheet of the hair follicle, red arrows to the hair channel. Scale bar in (A, B, D, H) 20 μm; in (C) 100 μm; in (E, F, G) 40 μm.

When skin samples of newborn and adult rats were compared, it became obvious that immediately after birth GS is strongly expressed in stratum basale of rat scalp and much less intense in dermal fibroblasts as shown by confocal laser scanning (CLS) micrographs of newborn rat scalp sections (white arrows Figure 3A, B). In hair follicles of newborn rat scalp, GS was prominent in the outer sheet and in the medulla (red arrows in Figures 3E–G). CLS microscopy of culture-activated fibroblasts isolated from young human skin showed strong reaction of GS in cytoplasm and especially nuclei of the cells (yellow arrows in Figures 3D, H).

**Figure 4:**
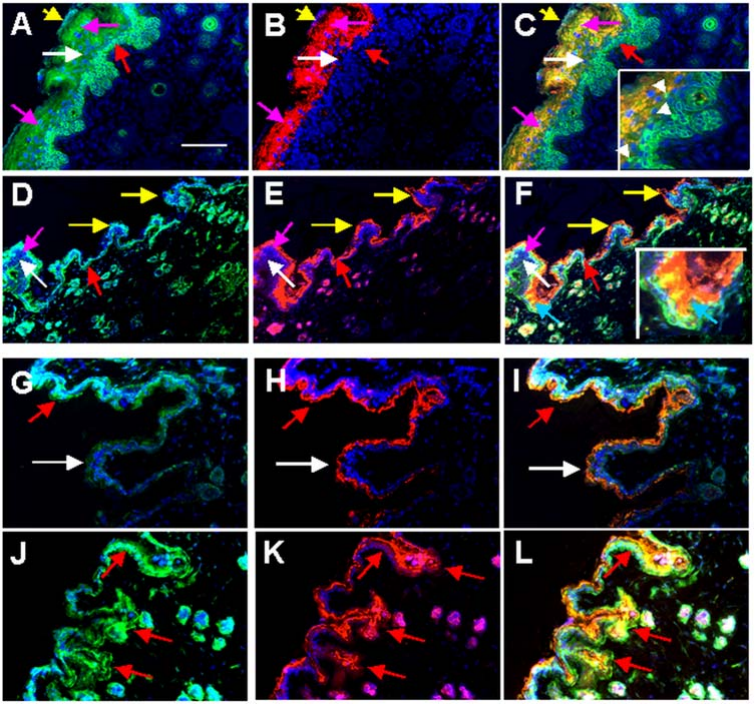
Age-dependent distribution of GS and GFAP in rat scalp. (A–C) Double labelling of newborn (2–3-day-old) rat scalp. (A) staining for GS (green, pAb), (B) staining for GFAP (red, mAb), (C) merge. (D–F) Double staining of adult rat scalp sections. (D) staining for GS, (E) staining for GFAP, (F) merge. The insert in (C) shows single keratinocytes strongly stained for GS. White arrowheads in the insert point to GS-positive cells migrating towards the outer layer of the skin. The insert in (F) shows the prominent differences in the ratio of GS and GFAP in neighboring regions of the epidermis of adult rat skin evidenced by green, yellow or orange staining. Red arrows in (A–F) point to stratum basale, white arrows to stratum spinosum, magenta arrows to stratum granulosum, yellow arrows to stratum corneum. (G–L) Co-expression of GS and GFAP in adult rat leg skin. (G–I) Double labelling of skin section from the lateral surface of adult rat leg skin. (G) staining for GS (green, pAb), (H) staining for GFAP (red, mAb), (I) merge. (J–L) Double staining of skin section from the medial surface of adult rat leg skin. (J) staining for GS, (K) staining for GFAP, (L) merge. White arrows in (G–L) point to the portion of the skin in which GS can be barely detected, whereas red arrows point to the portion of the skin in which GS is strongly expressed. Binding of specific antibodies was visualized with FITC-conjugated goat anti-rabbit IgG (A, D, G, J) and Cy3-conjugated goat anti-mouse IgG (B, E, H, K). Scale bar: (A–C) 10 μm; (D–F) 20 μm.

After few days, i.e., in 2–3-day-old rat scalp sections GS was found in all layers of epidermis. GS expression (green) was significantly increased in stratum granulosum (magenta arrows in Figure 4A–C) and was present in corneocytes in some regions of stratum corneum (yellow arrows Fig 4A–C) which at that time still contained many nuclei or nuclear remnants revealed by 4′,6 diamidino-2-phenylindole (DAPI) staining. At this age, co-localization of GS (green) with GFAP (red) in stratum corneum was less prominent than in stratum granulosum (cf. magenta and yellow arrows in Figure 4C). By contrast, in adult rat scalp staining intensity of GS at the interface of stratum granulosum and stratum corneum decreased (Figure 4D), whereas in the same region GFAP dominated (white arrows in merged Figure 4F). In adult rat scalp sections, the number of nuclear remnants in stratum corneum was strongly decreased compared to aged skin in humans. Though this type of co-localization of GS and GFAP was preserved in other skin samples of adult rats, for instance in leg skin (Figure 4G–L), the ratio between GS (green) and GFAP (red) seemed to vary slightly depending on whether samples from lateral (Figure 4G–I) or medial (Figure 4J–L) leg skin were stained. Thus, in the lateral surface of the leg skin many regions lacking GS (white arrows in Figure 4G–I) could be found, whereas in medial surface of the leg skin the GS/GFAP ratio was significantly increased (red arrows in Figure 4G–L).

#### Co-localization of GS and metallothionein in skin slices and cultured cells

**Figure 5:**
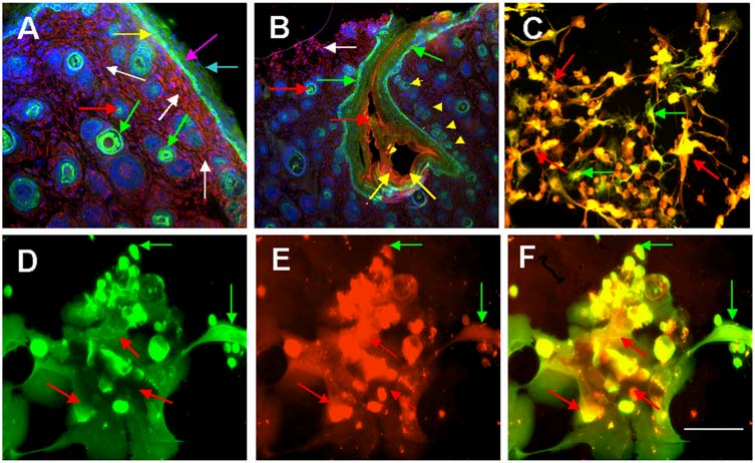
Co-expression of GS and metallothionein in rat skin sections and in cultured human keratinocytes and rat brain astrocytes. (A, B) Double-staining for GS (green) and MT (red) in newborn ratscalp sections. (B) Expression of GS (green) and MT (red) in rat scalp hair follicle. Cell nuclei in A–L are stained with DAPI (blue). (C) Co-expression of GS (green) and MT (red) by cultured newborn rat astroglial cells. (D–F) Expression of GS (D) and MT (E) and their co-localization (F, merge) in cultured human skin keratinocytes. GS was detected using rabbit antiserum and FITC-conjugated goat anti-rabbit IgG, whereas MT was detected using mAb MT and Cy3-conjugated goat anti-mouse IgG. Blue arrows point to stratum corneum, yellow arrows in (A) to stratum basale, magenta arrows to the keratinocytes forming the stratum granulosum, red arrows in A and B to the hair channel, green arrows to the outer sheet of the hair follicle, white arrows to fibroblasts; yellow arrowheads in (B) to sweet glands. Red arrows in (D–F) point to cells which predominantly express MT, green arrows in (D–F) point to cells which predominantly express GS. Scale bar: in (A, D, E, F) 10 μm; in (B, C) 20 μm.

The pattern of staining for metallothionein (MT) in newborn rat scalp (Figure 5A, B) was different from that of in human skin described previously [21]. In human skin, keratinocytes strongly expressing MT in all layers of epidermis exhibited widespread co-localization with GS. By contrast, in newborn rats, the only region of the epidermis reacting with mAb MT (red) was the stratum basale (magenta arrows in Figure 5A). Only in this layer of epidermis, GS (green) showed some co-localization with MT. In rat scalp skin dermis, GS and MT were localized in functionally different dermal cells. MT (red) was evenly distributed in GS-negative fibroblasts throughout the dermis (white arrows in Figures 5A, B), whereas GS was prominent in the outer sheet (green arrows in Figure 5A, B) and medulla of hair follicles (red arrows in Figure 5A, B). Though weakly, GS was also expressed in the sweat glands (yellow arrowheads in Figure 5B). MT and GS were also present in germinative epidermal cells located in the lower end bulb region of rat hair follicles (yellow arrows in Figure 5B).

When human keratinocytes were brought to culture, they maintained a strong staining for GS for 6 days (Figure 5D). Simultaneously, they expressed MT (Figure 5E) evidenced by overlapping yellow stain in merged micrograph (Figure 5F). However, different cells could be observed which expressed GS stronger than MT (green arrows in Figures 5D–F) and vice versa (red arrows in Figures 5D–F). The heterogeneity in the intensity of staining of GS and MT in neighbouring keratinocytes was comparable with that of rat astroglial cells (cf. Figures 5F and 5C). This fact further evidenced the specificity of antigen recognition by the anti-GS antisera and mAb against MT.

### Factors influencing GS expression and activity in skin

#### HaCaT cells

Regulatory features of GS were investigated in primary human keratinocytes and particularly in HaCaT cells, a spontaneously immortalized human keratinocyte cell line [33] in which the GS activity was only slightly higher compared to primary human keratinocytes. Thus, the specific activity of GS in primary human keratinocytes cultured for 24 h amounted to 4.3±1.8 mU/mg protein (Table 1), whereas the average GS activity in HaCaT cells amounted to 6.8±4.8 mU/mg protein (Table 1). difference between the primary cells and the HaCaT cells was statistically insignificant due to the high variation of the activity in this cell line.

**Table 1:** Effect of NH_4_Cl, LiCl and dexamethasone on the specific activity of GS in primary human keratinocytes and HaCaT cells.

Treatment^1^	Keratinocytes	HaCaT cells
	GS specific activity [mU/mg]	
Control	4.3±1.8^2^	6.8±4.8
NH_4_Cl (30 mM)	11.4±0.6*	65.1±6.2*
LiCl (50 mM)	7.1±1.6	7.7±1.4
dexamethasone (1 μM)	5.1±2.8	57.3±3.7*

1 Cells were incubated for 24 h at the indicated concentrations for the different stimulators.

2 Values represents means±SD of 3 to 8 determinations.

* statistically different from respective controls, p<0.01

#### Cell density/glutamine

**Figure 6:**
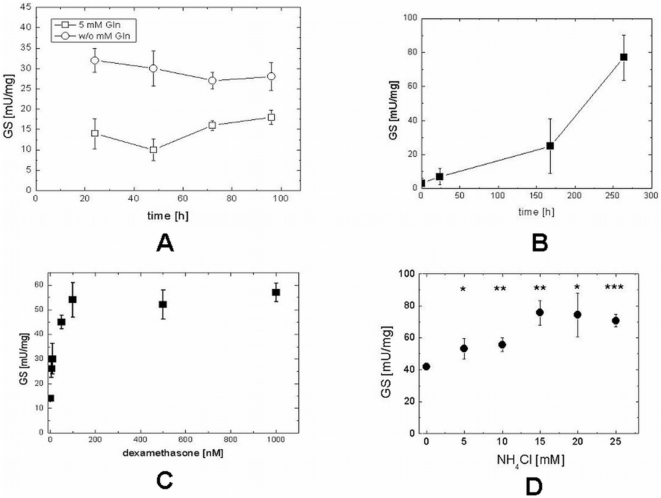
Regulatory features of GS in HaCaT cells. (A) Effect of Gln on the specific activity of GS. Cells were cultivated over 96 h with (squares) and w/o (circles) 5 mM Gln. Medium was renewed every day. Each point represents the mean±SD of 3 determinations for a representative culture. (B) Effect of cultivation time on the specific activity of GS. Cells were cultivated for the times indicated and then harvested. Culture medium was exchanged 3 times a week. Each point represents the mean±SD of 3 determinations for a representative culture. (C) Effect of dexamethasone on the specific activity of GS. Cells were incubated with the indicated concentrations of dexamethasone for 24 h. Each point represents the mean±SD of 3 determinations. (D) Effect of ammonium chloride on the specific activity of GS. Cells were incubated for 24 h in the presence of the indicated concentrations of NH_4_Cl. Each point represents the mean±SD of 3 determinations for a representative culture. Statistically significant from controls: *, p<0.05; **, p<0.01; ***, p<0.001.

Careful investigation of this variability revealed that GS activity in the growing HaCaT cell line was considerably influenced by the cell density as well as by the glutamine content of the culture medium. Other differences between the culture media seemed of less importance. When HaCaT cells were cultivated for 24 h in the absence of Gln, GS activity was approximately 2-fold higher than in the presence of 5 mM Gln (Figure 6A). While the higher activity in the absence of Gln remained nearly constant for 96h, the initially low activity in the presence of Gln slightly increased with time in parallel to the sustained cell growth under these conditions. Accordingly, when HaCaT cells were plated at low cell density, GS activity was very low (Figure 6B). During prolonged cultivation (>96 h), again, there was a steady increase in specific activity of GS as cell density increased. After 168 h, when cell layers had reached confluence and were highly packed, GS activity increased dramatically (Figure 6B) indicating that intensive cell-to-cell interactions are an important factor for high GS expression in this cell line.

#### Dexamethasone

As found with many cell lines [31], [34], [35], the synthetic glucocorticoid dexamethasone strongly (8-fold) induced the activity GS in HaCaT cells (Table 1). Examining of the concentration-dependency of this effect revealed that maximal induction in the HaCaT cells was reached above 0.1 μM dexamethasone (Figure 6C). Partially, this induction occurred at the transcriptional level, because GS mRNA determined by quantitative reverse transcriptase polymerase chain reaction (RT-PCR) increased 2.38-fold±0.38 (N = 3; p<0.05) after induction by 1 μM dexamethasone within 24 h (Table 1). Surprisingly, primary keratinocyte culture did not respond. Whether this may be due to the fact that keratinocyte growth medium contains hydrocortisone and, thus, primary cells might already reflect a somewhat induced state remains to be determined. Since hydrocortisone is vital for maintaining primary keratinocytes in culture, this hypothesis cannot directly be tested experimentally.

#### Ammonium ions

A rather unexpected finding obtained using HaCaT cells was that GS activity strongly increased in response to exposure to NH_4_^+^ ions. Depending on the specific activity of controls an increase in GS activity, up to approx. 9-fold, could be observed after the addition of 30 mM NH_4_Cl (Table 1). Figure 6D shows the concentration-dependence of this effect. Even in dense cultures showing a high control activity such as depicted in concentrations as low as 5 mM NH_4_Cl (Figure 6D) caused a significant increase in specific GS activity within 24 h. Generally, exposure to high concentrations of NH_4_Cl led to the highest GS activites around 80 mU/mg protein measured in HaCaT cell cultures. Even at concentrations as high as 30 mM NH_4_Cl no changes in cells size or shape were visible in bright field microscopy. In contrast to enzyme activity, the increase in GS mRNA over controls determined by quantitative RT-PCR under all conditions was insignificant. For instance, in the presence of 30 mM NH_4_Cl the fold increase of GS mRNA amounted only to 1.45±0.28 (N = 4). GS activity in primary human keratinocytes similarly responded to ammonium ions (Table 1), although the increase was less prominent compared to the cell line. Absence of alteration in molecular activity of the enzyme under these conditions (not shown) excluded posttranslational modification. These findings suggest a marked posttranscriptional influence of NH_4_Cl on GS, similar to that described in skeletal muscles [36].

#### Lithium chloride

In order to learn more about the possible involvement of β-catenin signalling in the regulation of GS in skin, we exposed cultured human keratinocytes and HaCaT cells to LiCl, a commonly used glycogen synthase kinase 3ß (GSK3β) inhibitor [37] that is able to activate β-catenin and to induce GS in hepatocellular populations [38], [31]. As shown in Table 1, LiCl failed to significantly induce GS in both types of cells, although activity values tended to be slightly increased. Thus, β-catenin signalling may be necessary, but not sufficient for reaching high levels of GS protein in keratinocytes.

## Discussion

### Cellular localization of GS in skin and its correlation with neural marker proteins

The present study demonstrates the previously unknown distribution of GS in human and rat skin. It shows unexpectedly large stores of GS in epidermal keratinocytes in human and rat skin sections. This study contradict to low activity of this enzyme in rat and murine skin homogenates demonstrated in very few reports elucidating the presence of GS in normal skin [14], [16]. The accumulation of GS in all epidermal cell layers was detected using mAB and pAb against GS. The authenticity of antigens recognized by pAb GS is confirmed also by heterogeneity in the intensity and pattern of GS staining between neighboring cells and regions of epidermis and dermis, as well as between cultured human keratinocytes, that might be due to differences in the individual rates of differentiation of these cells. The expression of GS throughout all layers of the epidermis suggests its importance for several skin-specific functions (see Table 2). Thus, the presence of GS in stratum basale suggests its involvement in sustained rejuvenation of keratinocyte population, its accumulation in stratum granulosum may indicate its involvement in the establishment of the epidermal permeability barrier, whereas its prominent expression in the outermost layer of the epidermis, the stratum corneum may hint at its necessity for the physical and chemical defense [39]. The loss of these functions as observed in inherited GS deficiency [17] could explain the severe skin pathology associated with this disease.

**Table 2:** Age- and region-dependent distribution of glutamine synthetase in human and rat skin sections.

	Human skin		Rat skin				
	Young foreskin	Aged temple	Newborn scalp skin	2-day-old scalp skin	2-year-old scalp skin	2-year-old leg skin, lateral area	2-year-old leg skin, medial area
**Stratum corneum**	++++	−	−	++	+	+	++
Stratum granulosum	++++	++++	+	+++	++++	++	++++
Stratum spinosum	+++	+++	+	+	++	++	++
Stratum basalis	+++	+++	++++	++++	++	++	++++

The cells were labelled with polyclonal antibodies against GS. The intensity of staining was estimated visually as absent (−), weak (+), moderate (++), strong (+++) or very strong (++++).

### GS and epidermal permeability barrier

GS is related to proteins involved in establishment of protective barriers against external signals at the blood-tissue interfaces in brain and liver. The present study shows that GS is essential also for establishment of epidermal permeability barrier. The large amount of GS throughout the epidermis and also in dermis is in agreement with the main function of skin to control the homeostasis of the organism. The intimate connection of Gln-enriched proteins to an epidermal barrier function has been proven in human skin diseases [40] and in transgenic mice overexpressing claudin-6, i.e., tight junction protein in the epidermis [41]. Therefore, keratinocytes of stratum granulosum carry a particular burden, because in this layer cells synthesize lots of proteins and other material important for terminal differentiation of keratinocytes into corneocytes and generation of the stratum corneum. Many of structural proteins involved in establishment of epidermal barrier for example involucrin, loricrin, and filaggrin, contain a disproportionally high percentage of Gln [41]–[44] and are increased during development of epidermal barrier [41], [45]. Thus, synthesis of skin proteins creates a strong demand for enhanced intracellular pool of Gln that most likely cannot be satisfied by diffusion of Gln from the blood through stratum basale and stratum spinosum. Consequently, high local production based on the predominant expression of GS in stratum granulosum and the adjacent part of stratum corneum may be of utmost importance to guarantee an optimal supply of Gln.

An epidermal barrier is in part formed by tight junction components which in adults are localized to a narrow zone of the granular cells [46]. Strong expression of GS in stratum granulosum and stratum corneum of young human skin and its redistribution into the stratum granulosum and inner layers of the epidermis in senile human skin shown in the present study might reflect its active involvement in protection provided by tight junctions. Indeed, GS has been shown to prevent disruption of the tight junctions by acetaldehyde, to maintain the transepithelial resistance and to decrease the paracellular permeability [28]. Furthermore, exposure of intestinal cell cultures to Gln influenced the expression of functional components of tight junctions, i.e., claudin-1, occludin, and ZO-1 protein [29].

The timing of GS expression in keratinocytes coincides with production of MT, another proliferation-inducing and barrier supporting factor [47]. Presence of MT in different cell types evidence their capacity to reflect the attack of immunologic stressors, oxidative stress and intoxication caused by heavy metals [48]. The co-localization of GS and MT has been shown also in functionally active astrocytes and activated HSC [7], [9], [21], [22]. In the light of elevated occurrence of cadmium in the environment and increased human use of this toxic heavy metal [49], a further role for MT in skin specific functions may be related to protection against cadmium-induced damage of tight junctions [50], [51]. After being trapped by metallothionein, ionic cadmium [Cd^2+^] lost its capacity to damage the tight junctions between LLC-PK1 cells [50]. Co-production of GS and MT by young human foreskin epidermal cells and especially by keratinocytes situated in the stratum basale of newborn rat skin is in accordance with literature data showing direct correlation between neural and non-neural cell proliferation rates and concentrations of MT [23] and GS [12], [22], [52].

Actin cytoskeleton plays an essential role nearly in all functions performed by skin, i.e., cell division [53], contraction [54], migration [55], [56] and stratification of epidermis [57]. SMAA, the marker protein of myofibroblasts, is also related to junction-associated proteins [58], [59]. In active states of the GFAP-positive HSC, mesangial cells, astrocytes characterized by increased capacity for proliferation and resistance to environmental insults SMAA is contracted to the perinuclear area [23], [24] similar to that of observed in keratinocytes. Simultaneous appearance of SMAA, GS and MT at functionally active states of astrocytes, HSC, mesangial cells and keratinocytes [21], [23], [24]. further emphasizes our hypothesis of the commonness of mechanisms operating at the border of different environments and tissues, in particular at the integumentum. Functional association between actin, tight junction, adherens junction proteins and GS were evidenced by reduced acetaldehyde-induced dissociation of occludin, zonula occludens-1, E-cadherin, and beta-catenin from the actin cytoskeleton when Caco-2 cells were exposed to Gln [28]. In this study, an indirect evidence of functional association of actin with mechanical barrier provided by tight junction [58]–[60] is presented by dense lining of actin-positive cells in stratum granulosum of young human foreskin. A week SMAA immunoreaction and moderate mRNA have been shown in a small number of keratinocytes in the interscapular region of intact newborn rat skin [61]. Furthermore, in a current study on adult murine skin we found that the number of SMAA-synthesizing keratinocytes was strongly dependent on the strain of mice and was sometimes lower than in human skin (unpublished results).

Epidermal keratinocytes are faced with the complex task of providing anti-cytotoxic and mechanical defense supported by SMAA cytoskeleton, as well as facilitated exchange with external environment and immune barriers supported by GFAP cytoskeleton. High levels of filamentous GFAP expression in differentiated astrocytes and non-differentiated HSC and mesangial cells were associated with cellular functions associated with facilitated transport and down-regulation or absence of GS, MT and SMAA [23], [24], [62]. Therefore, co-expression of GFAP and major histocompatibility complex II by keratinocytes [21] on the one hand, and of MT, SMAA and GS on the other hand, is a further evidence of complex tasks of keratinocytes to distinguish between external signals to be utilized from those to be blocked. Further studies are necessary to understand the enigma of appearance of SMAA, the marker protein of myofibroblasts and mesenchyma cells, in epidermal keratinocytes.

### Age associated changes of GS

The balance between growth and apoptosis of keratinocyte providing continuous renewal and resistance of stratum corneum to external signals is disturbed with age. The mechanisms involved in this process are not well understood. Our study shows that production of GS by skin is developmentally regulated and that the dynamics of postnatal differentiation of GS in human and rat skin is comparable with age-dependent changes in GS expression in human and rat skin (Table 2). Both in humans and rats aging of the skin led to accumulation of GS in stratum granulosum and downregulation of the enzyme in stratum basale. Presence of GS in newly formed corneocytes which at that time still contained many nuclei evidenced the importance of GS for functions performed by living cells. Likewise, in young foreskin which showed the smallest number of stratum corneum cell layers GS was found in all layers. By contrast, the stratum corneum of aged human temple skin which demonstrated multiple layers of cells in stratum corneum was devoid of GS. The data presented further suggest that GS is always associated with developing keratinocytes up to the stage of terminal differentiation of the corneocytes that results in programmed cell death partially resembling apoptosis [63], [64]. The differences observed in stratum corneum cell layers depending on location, age and among different individuals corroborate previous findings [26] demonstrating the smallest number of stratum corneum cell layers in genital skin compared to that of face and scalp.

In accordance with studies demonstrating age-dependent decrease in the permeability barrier of the skin and seized capacity of skin stem cells for repair with age [25], [65], [66], strong expression of GS in newborn rat skin stratum basale, consisting mainly of stem cells, slightly decreased in adult animals. Also the intense staining for GS throughout the epidermis of young human foreskin implies high potency of this skin region for proliferation and regeneration. The presence of β-catenin in inner layers (spinous and granular layers) of aged skin epidermis shown herein demonstrates the upkeeping of the stem cell pool in elderly [67]. Changes in the architecture of senile human skin were illustrated not only by absence of GS and thickening of stratum corneum but also by dense laying of keratinocytes intensely stained for GS in stratum granulosum. Whether the redistribution of GS from stratum corneum to stratum granulosum, a region enriched by tight junction-associated proteins [58], [46], reflects an age-associated dehydration leading to laxity and changes in the architecture of the skin [68] is a matter of future investigations.

Simultaneous presence of GS and β-catenin in keratinocytes of stratum basale and stratum spinosum and the presence of activated cytoplasmic and nuclear β-catenin in these cells shows similarities to the regulation of GS expression in hepatocytes. There, active Wnt/β-catenin signalling is required for initiating GS expression [31], [32], [38]. Our findings concerning co-localization of β-catenin and GS suggests that the mechanism of post differentiation patterning recently hypothesized for liver tissue [31] may likewise be relevant for skin. Briefly, post differentiation patterning suggests that β-catenin signalling acts on proliferating or differentiating cells imprinting them to express GS and rendering this expression to be regulated by various other signals. These features are then maintained in quiescent, post-mitotic cells even in the absence of continuous β-catenin signalling and allow extremely high expression levels of this enzyme [31]. Exactly this situation seems to occur when keratinocytes terminally differentiate in stratum granulosum where no activated β-catenin was found, but the highest concentration of GS is reached.

### Ammonium ions as regulators of GS activity

The regulatory mechanisms controlling the GS activity in skin present a mixture of mechanisms controlling this enzyme in inner organs with an unusual mode of regulation by ammonium ions, pointing to hidden mission of this enzyme in the integumentum. Liver and brain are the major ammonia consuming systems metabolizing it to Gln [69]. Among mechanisms triggering blood-brain barrier alterations in hepatic encephalopathy and its reestablishment after reversal of fibrosis, the central place is given to hemodynamic changes and circulating ammonia [70], [71]. GS prevents the entry of high amounts of ammonia from circulation to brain and thus, attenuates neurotoxicity. The changes in the distribution of this critical enzyme suggest that the glutamate-glutamine cycle may be differentially impaired in hyperammonemic states [72]. Both brain and liver reacted to hyperammonemia by down-regulation of GS [68], a response that was also found in cultured hepatocytes [3]. In contrast, skeletal muscles react to increase in blood ammonia level by up-regulation of GS [73]. In skeletal muscle, enzymatic activity (but not gene) was significantly up-regulated [36], [68], [73], [74]. In the hyperammonemic rat muscle ammonia levels were always increased and muscle Gln decreased, probably indicating glutamate consumption by enhanced Gln synthesis [75].

Recent studies showed that ammonia was present in gas emanated from the skin surface of healthy persons and patients with hepatic disease. The average level of ammonium ions removed via skin correlated with that of in blood and it reached the highest amounts in so-called skin gas collected from patients with hepatic disease [76]. This mode of elimination of circulating ammonia may contribute to and partially explain the large amounts of GS in the skin. Judging from the fact that erythema could be induced when highly ammoniacal urine was applied exclusively to scarified skin [77], the roots of the severe skin pathology in GS-deficiency [17] primary lay in deficit of Gln leading to reduced production of structural proteins. In the light of our results demonstrating the GS-activating effect of ammonia, the removal of circulating ammonia via skin presents a natural continuously acting mechanism of regulation of GS activity favoring the local skin requirements for Gln in normal physiological conditions as well as in pathologic situation. Large amounts of GS in keratinocytes in situ and in culture and over-activation of GS (but not mRNA) in cultured keratinocytes exposed to ammonium ions, allows to consider the skin as powerful ammonium detoxification pool which comes to action during hyperammonemia. On the other hand, it is well established that hyperammonemia may disrupt blood-brain barrier integrity [78]. Also Madin-Darby canine kidney cells responded to elevated extracellular NH_4_Cl concentrations by distinct alterations in the architecture and transepithelial transport properties, redistribution of occludin, a tight junction protein, and reorganization of the actin cytoskeleton [79]. Therefore, the continuous irrigation of normal epidermis with small amounts of circulating ammonia may play a positive role not only in the sense of activation of GS, but also in preservation of exchange between the skin and external world.

### Other factors regulating the GS activity

The presence of ornithine aminotransferase in skin [80] renders it possible that a similarly efficient production chain for Gln exists in epidermal keratinocytes as it is found in pericentral hepatocytes, the predominant site of Gln formation in the liver [31]. Nonetheless, further mechanisms seem to exist which can enhance GS activity, in order to match the need for an extraordinary high Gln synthetic capacity in certain skin layers. Regulation of keratinocytic GS by glucocorticoids and Gln shows many features known for other cells, e.g. astrocytes [81]–[84]. The same is true for regulation of GS by the density of keratinocytes. Naturally, high density of cultured keratinocytes provides higher level of GS, therefore of Gln, an amino acid known to induce the proliferation of different cell types. Also GS activity of liver-derived epitheloid cell lines and astrocytes is upregulated with increasing cell density and peaks in confluent cultures [34], [85].

Extrapolating the results obtained on cultured skin cells into in vivo conditions allows to propose that epidermal permeability barrier depends on Gln-induced expansion of keratinocytes that in turn increases the density of cell sealing and logically limits the paracellular permeability. The findings reported herein for keratinocytes contrast with those reported for dermal fibroblasts [86]. Thus, despite the presence of GS in many cell types of the skin, this organ does not seem to act uniformly with respect to GS expression and Gln synthesis, but rather shows considerable heterogeneity tailored to serve complex skin-specific functions.

### Concluding remarks

The present study comprises several experiments opening a new view on the physiological importance and multiple roles of GS in a complex series of events foreseen by an epidermal program aiming at the establishment of epidermal permeability barrier supported by sustained rejuvenation, differentiation, transepithelial resistance, detoxification, immune protection and cell-to-cell interactions. Since GS expression in skin is developmentally regulated, it may, thus, additionally be involved in mechanisms regulating the balance between cellular growth and programmed cell death.

Our study suggests that the apparently low activity of this enzyme in skin was previously underestimated resulting from measurements of average enzymatic activity in whole skin homogenates containing both enzymatically active and silent GS. These studies neglect the local skin-specific cellular functions and undervalued the capacity of keratinocytic GS for being modulated in health and disease. The results reported here in conjunction with literature data open new perspectives in the investigation of skin cell functions in healthy and pathological states. They call for further elucidation of the functional predestination of large stores of immunoreactive but enzymatically silent GS in epidermis particularly in stratum corneum as well as of regulatory mechanisms controlling its enzymatic activity, and especially of the role of circulating ammonia in establishment of the epidermal permeability barrier. Thus our results favor not only local specific functions of GS comprising the production of excess Gln for regional biosynthetic purposes, they also suggest a life-essential role of keratinocytic GS for whole organism in health and disease.

## Materials and Methods

### 

#### Reagents

Dexamethasone, Gln, penicillin, streptomycin, POD-extravidin, diaminobenzidine, aminotriazole, goat serum and Triton X-100 were products of Sigma (Taufkirchen, Germany). NH_4_Cl, ethylenediaminetetraacetic acid (EDTA), H_2_O_2_, Roti-Histokit, Tween 20 were purchased from Roth (Karlsruhe, Germany). Oligo(dT) primer, primer for GS and β-actin, Superscript II Reverse Transcriptase, SeeBlue® Pre-Stained protein standards were products of Invitrogen GmbH (Karlsruhe, Germany). The culture medium Dulbecco's modified Eagles medium (DMEM) and fetal calf serum (FCS) were purchased from GIBCO (Eggenstein, Germany). Keratinocyte growth medium and human epidermal growth factor (Clonetics, USA), trypsin and the Light Cycler FastStart DNA Master+SYBR Green I Kit were products of Roche Diagnostics GmbH (Mannheim, Germany). Vectashield mounting medium was purchased from Vector Laboratories (Burlingame, CA, USA). RNA isolation was performed using the Nucleo Spin RNA II Kit from Macherey-Nagel (Düren, Germany). Cell culture material was obtained from Techno Plastic Products AG (Trasadingen, Switzerland) as well as from Greiner Bio-One GmbH (Frickenhausen, Germany).

#### Antibodies

Monoclonal mouse anti-GS and monoclonal mouse anti-β-catenin antibodies were products of Becton, Dickinson and Company Transduction Laboratories (Heidelberg, Germany). Goat anti-mouse IgG F_ab_–POD fragment was purchased from Sigma (Taufkirchen, Germany). Biotinylated goat anti-mouse IgG-Fc antibody and sheep anti-mouse IgG F_ab_-AP were products of Chemicon International Ltd. (Hampshire, United Kingdom). Monoclonal anti-GFAP (clone GF 12.24) and monoclonal antibody against SMAA (anti-SMAA, clone ASM-1) were products of Progen Biotechnik GmbH (Heidelberg, Germany). The monoclonal antibody against Cd/Zn-inducible MT-1 was a generous gift of Prof. U. Weser (University of Tübingen, Germany). Polyclonal anti-GS antibody against pig brain GS was raised in the laboratory of Dr. Jaenicke [87]. Fluorescein isothiocyanate (FITC)-conjugated goat anti-rabbit IgG and Cy3-conjugated goat anti-mouse IgG were from Dianova Jackson Immuno-research (West Grove, USA).

#### Animals

New-born and adult Sprague-Dawley rats (250 to 300g) were obtained from Charles River (Sulzfeld, Germany) and maintained at the central animal facility according to institutional guidelines for ethical care of animals. Samples of rat skin were prepared from the scalp of newborn rats after extirpation of the brain which was used for the preparation of astroglial primary cultures.

#### Human skin

Samples of human foreskin were obtained from the Childrens Hospital of the University of Leipzig with consent from patients who underwent circumscission (phimosis) and samples of temple skin of 70-year-old male (healthy border of a basal cell carcinoma) from the Dermatology department of the University of Leipzig. All human skin samples were taken after written consent of the patients and in accordance with the principles and under the license of the Ethic Commission of the University of Leipzig.

### Cell culture

#### Primary keratinocyte culture

Primary normal human epidermal keratinocytes were isolated from the foreskins of 2–8 year old children according to the methods described by Zellmer and Reissig [88]. Briefly, the cells were seeded in Petri dishes and were grown in serum-free keratinocyte growth medium supplemented with 0.15 mM CaCl_2_, 0.1 μg/ml human epidermal growth factor, 0.5 μg/ml hydrocortisone, 5 μg/ml insulin, 30 μg/ml bovine pituitary extract, 50 μg/ml gentamycin, and 50 μg/ml amphotericin. Cultures were terminated at day 7 as described previously [21].

#### Keratinocyte cell line

HaCaT cells [33] were cultivated using DMEM medium supplemented with 2 mM glutamine, 10% FCS, 50 U/ml penicillin and 37.5 U/ml streptomycin in 75 cm^2^ cell culture flasks. For sub-cultivation the cells were incubated with Hanks balanced solution without calcium, supplemented with 0.05% EDTA for 20 minutes. Then, cells were harvested using a Hanks solution composed of 0.05% trypsin and 0.025% EDTA and suspended in supplemented DMEM.

#### Fibroblasts

Monolayers of normal human dermal fibroblasts from the foreskins of 2–8 year old children were obtained from explant cultures of de-epidermized skin and cultured in DMEM containing 10% heat-inactivated FCS (Gibco, Karlsruhe, Germany), 50 μg/ml L-ascorbic acid, 100 U/ml penicillin, and 100 μg/ml streptomycin sulphate, as previously described [89]. The fibroblast cultures were kept in an incubator with 5% CO_2_ in air (95% humidity) at 37 °C. Cultures were terminated at day 1 and 3 in culture as described previously [21].

#### Astroglial cells

Rat astroglia-rich primary cultures were prepared from brains of newborn animals as described elsewhere [90].

### Immunohistochemistry and Immunocytochemistry

#### Indirect double- and mono immunofluorescence studies

Unfixed 7 μm cryostat human and rat skin sections, cultured human keratinocytes and fibroblasts, as well as cultured rat astroglial cells were prepared as described previously [91]. After fixation in methanol (−20°C), the cells and tissue slices were washed with phosphate buffered saline (PBS) at room temperature (RT). In monolabelling studies, the sections and cells were exposed to pAb anti-GS (dilution 1∶100) only. In double-immunolabelling studies, mixtures of pAb anti-GS with mAb against GFAP (dilution 1∶100) or mAb against Cd/Zn-inducible MT-1 (1.7 μg/μl, dilution 1∶100) [7], [23], [92], or mAb against SMAA (dilution 1∶100) were applied. After 2 h incubation at RT, specimens were washed and exposed for 1 h at RT to a mixture of the secondary antibodies: FITC-conjugated anti-rabbit IgG (dilution 1∶100) and Cy-3 conjugated anti-mouse IgG (dilution 1∶800). All antibodies were diluted with PBS. Subsequently, the cells and tissues were washed with PBS containing Triton X-100 and mounted in Vectashield mounting medium with or w/o DAPI.

#### Immunochemical detection of GS and β-catenin

Human skin samples were stored at −80 °C. Slices were cut and fixed in 3.5% paraformaldehyde immediately before staining. For the detection of β-catenin the tissue slices were permeabilized with 0.1 M Tris-borate buffer (pH 7.4) containing 0.2% Tween20 and the endogenous peroxidase activity was destroyed by incubation with 0.3% H_2_O_2_. The slices were incubated with monoclonal mouse anti-β-catenin antibody over night. After washing the secondary biotinylated goat anti-mouse IgG-Fc antibody was added for 1 h at RT followed by addition of the POD-Extravidin complex for 30 min. Staining was performed in 0.1 M Tris-HCl buffer (pH 7.6), composed of 0.05% diaminobenzidine, 2% aminotriazole and 0.033% H_2_O_2_. Finally the slices were dehydrated and embedded using Roti-Histokit. For the detection of GS the tissue slices were fixed and permeabilized as described for β-catenin staining. Non-specific binding sites were blocked with 10% goat serum and the slices were incubated with monoclonal mouse anti-GS antibody at 4°C over night. Subsequently the slices were washed and incubated with a goat anti-mouse IgG F_ab_–POD fragment. Staining and embedding was performed using Roti-Histokit. Nuclei were stained by immersion in Haemalaun solution for 2 min followed by washing for 10 min with tap water.

### Microscopy

#### Bright field microscopy

POD-labelled sections were visualized using a Leica DM5000 B microscope (Leica, Bensheim, Germany) equipped with a HCX PlanApo 40×/0.75 objective and a Leica FX 350 camera using the LAS software of Leica.

#### Fluorescence microscopy

FITC-conjugated anti-rabbit IgG-labelled and Cy-3 conjugated anti-mouse IgG-labelled sections and cells were examined using:

1. Wide-field fluorescence microscope BX 51 (Olympus Optical Co. Europe, Hamburg, Germany) with UplanFI 20×/0.5 Ph1, UPlanFI 10×/0.30 Ph1, and UPlanFI 40×/0.75 Ph2 objectives. Images were taken with the digital camera F-View II (Soft Imaging System, Leinfelden-Echterdingen, Germany) and data were processed with AnalySIS DOKU® (Soft Imaging System).
2. Confocal laser scanning microscope Leica SL equipped with an argon/krypton laser (Leica Lasertechnik, Heidelberg, Germany). To improve the signal-to-noise ratio, 8 scans of one focal plane were averaged. The confocal image data were obtained using a PLANAPO 63×, 1.32 NA oil immersion objective and confocal laser scanning microscope software (Leica Lasertechnik), and processed with Adobe Photoshop 7.0 software (Adobe Systems Inc., Mountain View, CA, USA).

### Determination of glutamine synthetase activity

Specific GS activity was determined by the method of Levintow [93] with minor modifications described by Gebhardt and Williams [94]. Briefly, 7.5×10^6^ cells were seeded into 6 well plates and cultivated in 1 ml medium at 37°C and 5% CO_2_. After washing with Hanks balanced solution the cells were harvested using a rubber police man, disrupted by sonication (Sonoplus, Bandelin, Germany) and the protein concentration was determined according to Bradford [95] in order to calculate the specific GS activity.

### Quantitative PCR

Cells were washed twice with Hanks balanced salt solution. Total RNA was isolated using the Nucleo Spin RNA II Kit according to the manufacturer description which included DNase I digestion. One μg of total RNA was reverse transcribed using oligo(dT) primer and Superscript II Reverse Transcriptase. The cDNA was diluted 1/10 and 3 μl were used for amplification of GS and β-actin as internal control using the Light Cycler FastStart DNA MasterPLUS SYBR Green I Kit. The following primers were used for GS: 5′-CACTACCGGGCCTGCTTGTAT-3′ (forward primer) and 5′-GCATGGCCTTGGTGCTAAAGTTG-3′ (reverse primer), for β-actin: 5′-AGAAAATCTGGCACCACACC-3′ (forward primer) and 5′-CTCCTTAATGTCACGCAC-GA-3′ (reverse primer). The PCR reactions were performed in duplicate in 20 μl volume containing 3 μl cDNA, 0.5 μM of each primer, and 4.0 μl 5×Master Mix. PCR products were quantified using a Light Cycler 2.0 (Roche Diagnostics GmbH, Mannheim, Germany) according to the following protocol: after heating for 10 min at 95°C, amplification was performed by 40 cycles of 3 s at 94°C, 5 s at 56°C and 20 s at 72°C, followed by a melting curve analysis. The fold induction of GS mRNA was calculated using the Relative Quantification Method of the Light Cycler 4.05 software (Roche Diagnostics GmbH).

### Statistical evaluation

Enzymatic determination or quantitative RT-PCR measurement were repeated independently at least 3 times as stated in the legends of figures and tables. Data reported represent means±standard deviation (SD). Statistical evaluation was performed using Student's t-test.
