# Correction: Keratinocytes as Depository of Ammonium-Inducible Glutamine Synthetase: Age- and Anatomy-Dependent Distribution in Human and Rat Skin

**DOI:** 10.1371/annotation/fa1fbbb8-5dca-4afb-a5ee-c7eb3f5c45bc

**Published:** 2009-03-16

**Authors:** Lusine Danielyan, Sebastian Zellmer, Stefan Sickinger, Genrich V. Tolstonog, Jürgen Salvetter, Ali Lourhmati, Dieter D. Reissig, Cristoph H. Gleiter, Rolf Gebhardt, Gayane Hrachia Buniatian

The 10th author's affiliation is incorrect. The affiliation should be institution 1: Department of Clinical Pharmacology, University Hospital of Tübingen, Tübingen, Germany.

